# Restudy of malformations of the internal auditory meatus, cochlear nerve canal and cochlear nerve

**DOI:** 10.1007/s00405-014-2951-4

**Published:** 2014-03-06

**Authors:** Youjin Li, Jun Yang, Jinfen Liu, Hao Wu

**Affiliations:** 1 Department of Otorhinolaryngology, Children Medical Center, Shanghai Jiaotong University School of Medicine, Shanghai, 200127 China; 2 Department of Otorhinolaryngology-Head and Neck Surgery, Xinhua Hospital, Shanghai Jiaotong University School of Medicine, Shanghai Jiaotong University School of Medicine Ear Institute, Kong Jiang Rd 1665, Shanghai, 200092 China; 3 Department of Pediatric Institute, Children Medical Center, Shanghai Jiaotong University, Shanghai, 200127 China

**Keywords:** Internal auditory meatus, Cochlear nerve canal, Cochlear nerve, Classification

## Abstract

The present study aims to restudy the correlation between the internal auditory meatus (IAM), the cochlear nerve canal (CNC), the cochlear nerve (CN) and inner ear malformations. In this retrospective study design, the abnormal diameter of the IAM, CNC and CN in patients with any kind of inner ear malformations was evaluated using multi-slice spiral computed tomography (MSCT) (37 patients) and magnetic resonance imaging (MRI) (18 patients). Of 37 MSCT-diagnosed patients, 2 had IAM atresia, 11 IAM stenosis, 22 enlarged IAM, and 2 normal IAM with an abnormal CN. MRI diagnoses of 18 patients revealed 8 cases of aplastic CN, 6 hypoplastic CN, and 4 normal CN. CNC stenosis was associated with CN hypoplasia (*P* < 0.001). Patients with absent or stenotic IAM had less CN development than those with normal or enlarged IAM (*P* = 0.001). We propose a modification of the existing classification systems with a view to distinguishing malformations of the IAM, CNC and CN.

## Introduction

The prevalence of congenital sensor neural hearing loss (SNHL) is approximately 1 in every 650 newborns [1]. 50 % of the congenital SNHL cases are caused by environmental exposures during pregnancy, while the other half by genetic mutations [2]. Diagnostic imaging has documented that 26 % of the cases have varying degrees of inner ear malformations, 65 % of which are bilateral [3]. Cochlear implantation (CI) is an option for the treatment of congenital SNHL. However, CI does not fit all types of inner ear malformations. A scientific classification system is needed to identify the cases suitable for CI.

Jackler et al. [4] was the first to put forward the classification system, which was later revised by Sennaroglu et al. [5] who classified the internal auditory meatus (IAM) malformations as absent, narrow, or enlarged, yet without taking into consideration the presence or absence of the cochlear nerve (CN). Casselman’s classification system [6] identified two types of IAM stenosis using HRCT and MRI: the absence of the vestibulocochlear nerve and the undeveloped or underdeveloped cochlear branch. However, his classification system is based solely on IAM stenosis to the exclusion of the cases where the CN is malformed but the IAM is normal or enlarged.

To address the above-mentioned problems, the present study sets out to explore a more specific evaluation framework for classifying congenital malformed IAM, cochlear nerve canal (CNC) and CN.

## Materials and methods

### Participants: pediatric patients with congenital SNHL

The participants (*n* = 860) included infants and children diagnosed with SNHL from February 2005 to January 2010 at the Department of Otolaryngology, Shanghai Children’s Medical Center, affiliated with Shanghai Jiaotong University School of Medicine. Congenital inner ear malformations were observed in 223 ears of 125 patients, 27 with unilateral malformations and 98 bilateral. 35 patients, aged between 6 months and 6 years had IAM malformations, had IAM malformations and 2 had normal IAM with an abnormal CNC or CN diameter. Excluded from the present study were the patients with the following cases: only external or middle ear malformations, a history of external or middle inner ear surgery, trauma, or temporal bone fracture.

### Auditory assessment and imaging

#### Auditory assessment

1. Acoustic immittance measurements (MEA Middle Ear Analyze, Madsen Zodiac 901, Denmark): measurements were carried out in a soundproof room, using a probe tone of 226 Hz with an additional tone of 1,000 Hz administered to patients under the age of 1.
2. Auditory brainstem response (ABR) test (Auditor Brainstem Response instrument, ICS CHARTR EP, USA): the ABR test was performed after sedation with oral or intramuscular sedatives. The normal hearing indicator was an ABR V wave response threshold ≤35 dB nHL with a 2–4 kHz range that was administered using air conduction (Note: there is no combination of external and middle ear malformation in this series).
3. Classification of hearing impairment: in accordance with 1981 American Speech–Language–Hearing Association recommendations for grading hearing, impairment, an ABR V wave response threshold >35 dB nHL served as the hearing loss standard. Hearing impairment was classified as follows: mild (26–40 dB), moderate (41–55 dB), moderately severe (56–70 dB), severe (71–90 dB), and profound (≥91 dB).

#### Multi-slice spiral computed tomography (MSCT) and MRI of the temporal bone

All CT examinations were performed with MSCT (16 slices, General Electric, Milwaukee, WI, USA). The supraorbital line served as the scanning range baseline, followed by scanning from the base of the external ear canal to the upper edge of the talus cone. The coronal reconstruction was parallel to the posterior wall of the maxillary sinus baseline, and extended from the posterior edge of the temporomandibular joint fossa to the anterior wall of the sigmoid sinus. Slice thickness, voltage and current were 1.00 mm, 120 kV, and 230 mA, respectively. The coronal reconstruction was performed at 1.00 mm thicknesses.

IAM widths were categorized as: normal = 2–8 mm; narrow <2.0 mm or an anteroposterior/ventrodorsal distance <2.0 mm; and enlarged >8 mm.

The diameter of the CNC is defined as the largest width between the inner walls of the modiolar base, as determined with horizontal sections of MSCT. Based on our results of CNC measurements in 52 ears (excluding one case of Michel malformation), the possibility of cochlear nerve abnormality should be considered if the diameter of the CNC <1.4 mm, as previously reported [7].

All MRI examinations were performed with a 1.5-T MR unit (Signa, General Electric, Milwaukee, WI, USA) with a head coil. Continuous scanning included an axial 3-D T2-weighted fast spin-echo sequence (using DRIVE: driven equilibrium). The scan thickness was 0.8 mm, scan aperture was 16–20 cm, TR was 7.9 ms, TE was 4.2 ms, array size was 320 × 256, and scan time was 240 s. MR source data were processed on an off-line workstation (Advantage Windows 4.2, General Electric, Milwaukee, WI, USA) with maximum intensity projection algorithm. Oblique sagittal images of nerve fibers in the IAM were reconstructed from perpendicular orientations of IAM to corresponding nerves. Four distinct nerves [the facial nerve (FN), CN, and the superior and inferior branches of the vestibular nerve] were detected at IAM’s lateral surfaces. Inner ear malformations were categorized as cochlear, vestibular, IAM, semicircular canal malformations, and vestibular aqueduct abnormalities. Sennaroglu’s et al. [5] vestibulocochlear malformation classification system was used to classify cochlear malformations (in descending order of severity), such as Michel malformation, cochlear aplasia, common cavity malformation, incomplete partition type I (IP-I, cystic vestibulocochlear malformations), cochlear hypoplasia, and incomplete partition type II (IP-II, Mondini deformity). Based on oblique sagittal reconstructions of MRIs, we defined CN hypoplasia as the CN with its diameter smaller than that of the FN, and CN aplasia as the CN not detected with MRI, similar to Kim’s definitions [8].

Our analyses involve the following aspects: (1) malformations of the inner ear and IAM evident by MSCT; (2) measurements of the CNC using MSCT; (3) CN hypoplasia evident by MRI; (4) relationships between IAM stenosis, CNC stenosis, and CN hypoplasia or aplasia.

### The M(C)ND classification system

We developed a M(C)ND classification system for describing anatomical variations of the IAM, CNC, CN, where:

- M represents IAM (0, IAM atresia; 1, IAM stenosis; 2, normal or enlarged IAM).
- C represents CNC (0, CNC atresia; 1, CNC stenosis; 2, normal or enlarged CNC; X-CNC not checked or unable to be checked).
- N represents CN development (0, aplasia CN; 1, CN hypoplasia; 2, normal CN; X-CN, not checked).
- D represents other inner ear deformities (0, severe malformations including Michel malformation, cochlear aplasia, common cavity, incomplete cochlear partition type I; 1, less severe malformations including cochlear hypoplasia, incomplete cochlear partition type II, enlarged vestibular aqueduct, simple vestibular malformations, simple semicircular canal malformations; 2, absence of cochlear–vestibular malformations).

### Data analysis

SAS 9.2 was used for the calculation of mean values, standard deviations, frequencies and percentages. Differences in the occurrence of CN hypoplasia for different IAM and CNC developmental grades were compared with Fisher’s exact test. Pearson correlation coefficients were calculated for different grades of CN development and CNC diameters.

## Results

### Auditory test

Of 37 patients (53 ears) with IAM malformations and normal IAM with an abnormal CN, moderately severe hearing loss was documented in 4 ears (7.5 %), severe in 18 ears (34 %), and profound in 31 ears (58.5 %).

### Imaging study

**Fig. 1:**
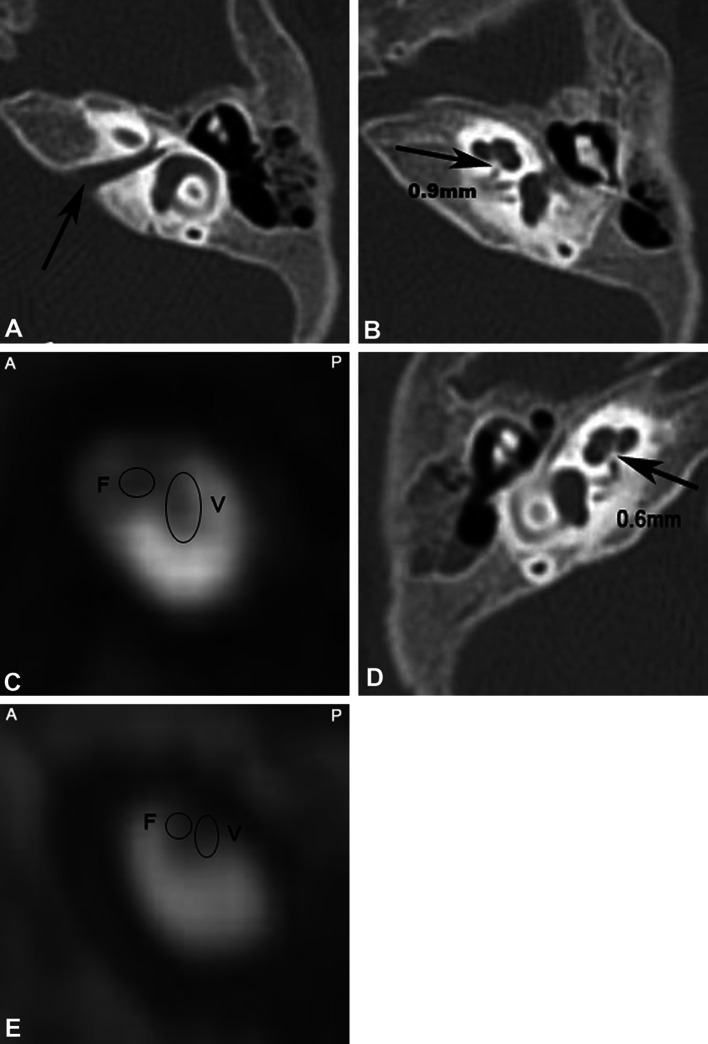
Stenosis of bilateral IAMs with an undetected CN. Male, 4 years old. Total hearing loss in the right ear, moderately severe hearing loss in the left ear. **a**, **b**, **d** CT shows bilateral stenosis of IAM, normal development of the cochlea (*arrow*). The diameters of the CNC are 0.9 and 0.6 mm. **c**, **e** Undetected CN with oblique sagittal reconstruction of MRI

Based on our proposed M(C)ND classification system, MSCT and MR imaging identified IAM malformations and normal IAM with an abnormal CN in 53 ears of 37 patients, as shown in Table 1. IAM stenosis was observed in 18 ears of 11 patients, and the IAM was absent in 3 ears of 2 patients. MR examinations were carried out in eight patients with IAM stenosis. The CN was not detected in four of these patients (6 ears) while CN hypoplasia was detected in the other four patients (6 ears). Bilateral IAM stenosis with an undetected CN was found in one case (Fig. 1).

**Table 1:** Malformations of the IAM based on the M(C)ND classification system (*n* = 37 patients)

Case no.	Side	M	C	N	D	Hearing level of SNHL	CI
1	R	M0	Cx	N0	D0 (Michel)	Profound	
	L	M0	Cx	N0	D0 (common cavity)	Profound	
2	R	M2	C1	N1	D0 (common cavity)	Severe	Yes
	L	M0	C0	N0	D0 (cochlear aplasia)	Profound	
3	R	M1	C0	N1	D0 (cochlear aplasia)	Profound	
	L	M2	C2	N2	D2	Normal	
4	R	M1	C1	Nx	D1 (vestibule/SCC)	Profound	
	L	M1	C1	Nx	D1 (vestibule/SCC)	Profound	
5	R	M1	C1	N0	D2	Profound	Yes
	L	M1	C1	N0	D2	Profound	
6	R	M1	C1	N0	D2	Profound	
	L	M1	C1	N0	D2	Moderately severe	
7	R	M1	C1	N1	D2	Severe	
	L	M1	C1	N1	D2	Severe	
8	R	M1	C1	Nx	D2	Severe	
	L	M1	C1	Nx	D2	Severe	
9	R	M1	C0	N0	D2	Profound	
	L	M2	C2	N2	D2	Normal	
10	R	M2	C2	N2	D2	Normal	
	L	M1	C1	N0	D2	Profound	
11	R	M1	C1	Nx	D2	Severe	
	L	M1	C0	Nx	D2	Profound	
12	R	M2	C2	N2	D2	Normal	
	L	M1	C1	N1	D2	Severe	
13	R	M1	C1	N1	D2	Moderately severe	
	L	M1	C1	N1	D2	Severe	
14	R	M2	C1	Nx	D1 (IP-II)	Severe	
	L	M2	C2	Nx	D2	Normal	
15	R	M2	C2	Nx	D1 (IP-II)	Severe	
	L	M2	C2	Nx	D2	Normal	
16	R	M2	C2	Nx	D1 (IP-II)	Moderately severe	
	L	M2	C2	Nx	D2	Normal	
17	R	M2	C2	Nx	D2	Normal	
	L	M2	C2	Nx	D1 (IP-II)	Severe	
18	R	M2	C2	Nx	D1 (IP-II)	Moderately severe	
	L	M2	C2	Nx	D2	Normal	
19	R	M2	C2	Nx	D1 (IP-II)	Severe	
	L	M2	C2	Nx	D2	Normal	
20	R	M2	C1	N2	D1 (IP-II)	Severe	
	L	M2	C2	N2	D2	Normal	
21	R	M2	C2	Nx	D2	Normal	
	L	M2	Cx	Nx	D0 (IP-I)	Profound	
22	R	M2	C2	Nx	D0 (IP-I)	Profound	
	L	M2	C2	Nx	D2	Normal	
23	R	M2	Cx	Nx	D1 (cochlear hypoplasia)	Profound	
	L	M2	C2	Nx	D2	Normal	
24	R	M2	C0	N0	D0 (common cavity)	Profound	Yes
	L	M2	C0	N0	D0 (common cavity)	Profound	
25	R	M2	C0	Nx	D0 (common cavity)	Profound	
	L	M2	C2	Nx	D2	Normal	
26	R	M2	C0	Nx	D0 (common cavity)	Profound	
	L	M2	C0	Nx	D0 (common cavity)	Profound	
27	R	M2	C0	N1	D0 (common cavity)	Profound	
	L	M2	C2	N2	D2	Normal	
28	R	M2	C0	N0	D0 (common cavity)	Profound	
	L	M2	C0	N0	D0 (common cavity)	Profound	
29	R	M2	C0	Nx	D0 (common cavity)	Profound	
	L	M2	C2	Nx	D2	Normal	
30	R	M2	C0	Nx	D0 (common cavity)	Profound	
	L	M2	C2	Nx	D2	Normal	
31	R	M2	C0	Nx	D0 (common cavity)	Profound	
	L	M2	C0	Nx	D0 (common cavity)	Profound	
32	R	M2	C0	Nx	D0 (common cavity)	Severe	
	L	M2	C0	Nx	D0 (common cavity)	Profound	
33	R	M2	C2	N2	D1 (enlarged vestibular aqueduct) (EVA)	Severe	Yes
	L	M2	C2	N1	D1 (enlarged vestibular aqueduct) (EVA)	Severe	
34	R	M2	C2	N2	D1 (vestibule/SCC)	Profound	
	L	M2	C2	N2	D1 (vestibule/SCC)	Profound	
35	R	M2	C2	N2	D2	Severe	
	L	M2	C2	N2	D2	Severe	
36	R	M2	C1	N0	D2	Profound	
	L	M2	C2	N2	D2	Normal	
37	R	M2	Cx	N0	D1 (cochlear hypoplasia)	Profound	
	L	M2	C2	N2	D2	Normal	

*IP-I* incomplete partition type I, *IP-II* incomplete partition type II (Mondini deformity), *SCC* semicircular canal

**Fig. 2:**
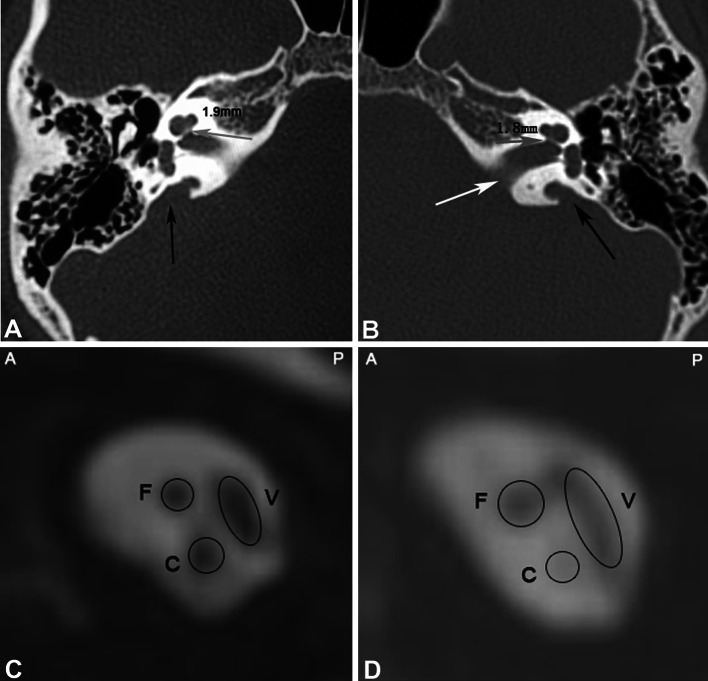
Enlargement of bilateral IAMs with CN hypoplasia. Male, 6 years old. Bilateral severe hearing loss. **a**, **b** Enlargement of the IAM, binaural enlarged vestibular aqueducts. The CNC on the right side is 1.9 mm (**a**), whereas 1.8 mm on the left side (**b**). **c**, **d** Oblique sagittal reconstruction of MRI imaging shows normal development of the right CN, FN and vestibular nerve in the IAM (**c**); the diameter of the left CN is smaller than that of the FN, suggesting CN hypoplasia (**d**)

**Fig. 3:**
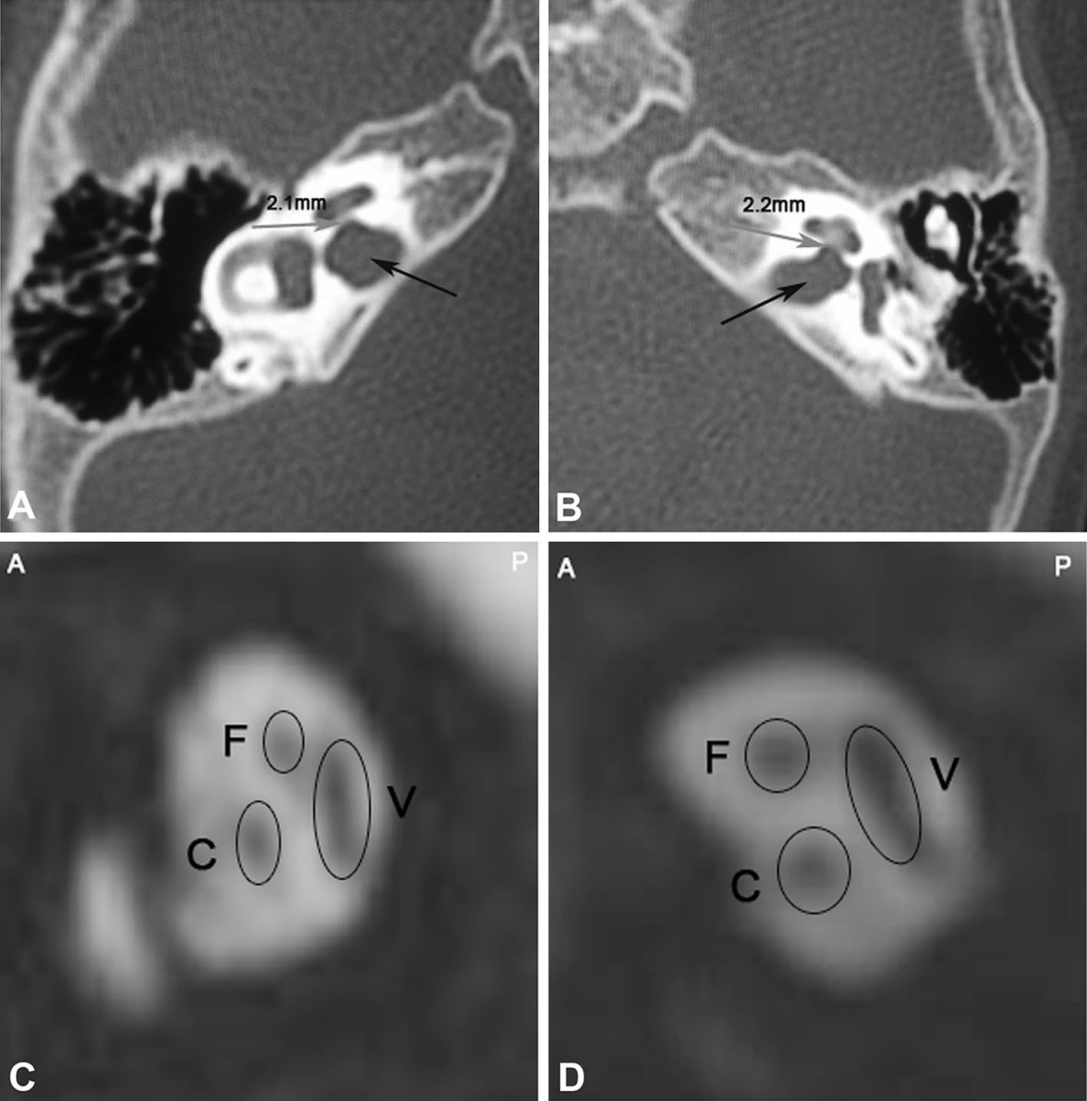
Enlargement of the IAM, with normal CN. Female, 2 years old. Bilateral severe hearing loss. **a**, **b** Axial CT shows an enlarged IAM. The CNC on the right side is 2.1 mm (**a**); 2.2 mm on the left side (**b**). **c**, **d** Oblique sagittal reconstruction of an MRI image shows normal development of the facial nerve in the IAM, the vestibular nerve, and the CN

IAM enlargement was detected in 22 patients (30 ears) by MSCT. Among ten ears with IAM enlargement, the CN was undetected in two ears (1 patient) while CN hypoplasia was detected by MRI in two ears (2 patients). Figure 2 shows bilateral IAM enlargement with CN hypoplasia, and Fig. 3 shows enlargement of the IAM with normal CN.

**Fig. 4:**
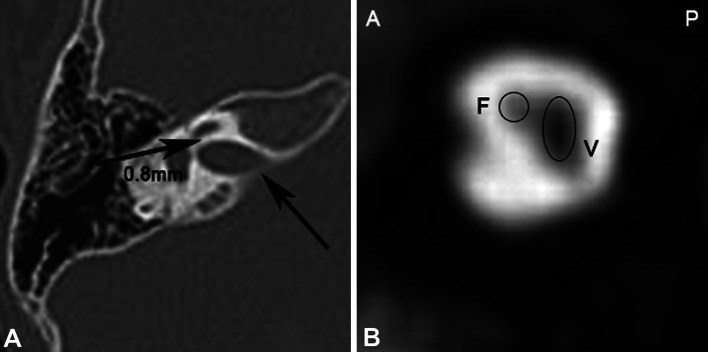
Normal IAM with an aplastic CN. Female, 2 years old. The right ear has total hearing loss and the left ear has normal hearing. **a** Axial CT shows a normal IAM. The diameter of the CNC is 0.8 mm (*arrow*). **b** Oblique sagittal reconstruction of an MRI shows the FN and vestibular nerve in the IAM. The CN is absent

MSCT imaging identified normal IAM and cochlear hypoplasia in one patient (1 ear). However, MRI did not detect the CN in the ear with cochlear hypoplasia (Fig. 4). Isolated aplastic CN was observed in one patient (1 ear), based on the MRI result.

Of 37 patients (53 ears) with IAM malformation and normal IAM with an abnormal CN, the CNC was not detected in 22 ears. The CNC diameter of 32 ears (0.4–2.3 mm; 1.30 ± 0.11 mm) with SNHL was smaller than that of 20 ears (1.4–2.3 mm; 1.83 ± 0.05 mm) without SNHL (*P* < 0.001). We considered that CNC diameters <1.4 mm were classified as stenotic. MR images were available for 18 patients in which CN abnormalities were identified in 22 ears, including aplasia in 14 ears and hypoplasia in 8 ears. The CNC diameter of the 22 ears (0.66 ± 0.13 mm) with CN developmental abnormalities was smaller than that of the ears with normal CN (1.78 ± 0.10 mm, *P* = 0.037) (14 ears).

CN hypoplasia was associated with an absent or stenotic IAM, compared to normal or enlarged IAM (*P* = 0.001) (Table 2). The diameter of the CNC with aplastic or hypoplastic CN was smaller than that of the CNC with a normal CN (Spearman’s *r* = 0.693; *P* < 0.001; 36 ears) (Table 3).

**Table 2:** Incidence of hypoplastic and normal CN in the IAM with differential developments (*n* = 36 ears)

IAM	CN hypoplasia (ears)	CN normal (ears)	*X* ^*2*^ value	*P* value
Absent	3	0	16.72	0.001
Narrow	12	0		
Normal	3	8		
Enlarged	4	6		

*CN* cochlear nerve, *IAM* internal auditory meatus

**Table 3:** Relationship between diameters of the CNC and differential developments of the CN (*n* = 36 ears)

CN	CNC average diameter (ears)		*X* ^2^ value	*P* value
	<1.4 mm	≥1.4 mm		
Non-developed	14	0	28.432	<0.001
Hypoplasia	8	0		
Normal	2	12		

*CNC* cochlear nerve canal, *CN* cochlear nerve

Of the 37 patients with IAM malformation and normal IAM with an abnormal CN, 4 underwent CI.

## Discussion

Hereditary deafness accounts for more than 50 % of cases with severe and profound congenital hearing loss, approximately 70 % of which cannot be categorized with existing classification schemes [2]. Normal inner ears are evident in the CT and MRI images of 70–80 % of patients with congenital SNHL while bony malformations are evident in the remaining minority of patients [5]. Congenital IAM malformations are classified as atretic, narrow, enlarged, or with abnormal orientations. Narrow IAMs are usually associated with CN and/or FN anomalies that could lead to identifiable symptoms, whereas IAMs that are wider than 8 mm are unlikely to be recognized as abnormal and are asymptomatic [9–11]. IAM stenosis accounts for about 12 % of all congenital temporal bone malformations and is associated with aplastic vestibulocochlear nerves [12]. In our study, IAM atresia (2 patients; 3 ears), IAM stenosis (11 patients; 18 ears), and IAM enlargement (22 patients; 30 ears) were observed in 35 patients with IAM malformations (51 ears). Two additional patients (2 ears) had normal IAMs with MSCT. Cochlear hypoplasia and an absent CN were found in MRI in one patient (case 37, right ear) with normal IAM. An isolated aplastic CN was observed in another patient (case 36, right ear).

### IAM

Inner ear development begins at the 22nd day of fetal development, with maturation at 8–16 weeks and ossification at 16–24 weeks [13]. Jackler et al. [4] proposed that arrested development at various stages of embryonic development can result in distinct inner ear malformations.

The vestibulocochlear nerve develops during the third week of pregnancy. During the ninth week, the cartilaginous IAM gradually develops in synchrony with the vestibulocochlear nerve’s development [14]. Some authors hold that absence of the vestibulocochlear nerve causes IAM aplasia or stenosis, while others believe that nerve development precedes cartilage development [15]. IAM stenosis could occur at various anatomical sites according to the stage of embryonic or childhood development, and could be most severe when there are concurrent severe inner ear malformations [16]. In our study, multiple concurrent inner ear malformations were found in 36 patients. Cochlear aplasia and common cavity malformations had greater severity in patients with IAM fundus stenosis than in patients with IP-II and vestibular semicircular canal malformations. Aplastic and stenotic IAMs had reduced CN development compared to patients with normal or enlarged IAMs.

### CNC

The CNC is considered a measurable and sensitive indicator of CN malformation. Moreover, CNC abnormalities may be a subtype of cochlear malformation [17]. Therefore, measurement of CNC diameters is valuable for identifying CN hypoplasia or aplasia. However, standardized diagnostic criteria for the CNC have not been established. Investigators have variably proposed CNC <1.4, <1.5, or <1.7 mm as cutoffs for CN hypoplasia [7, 18–21]. In the present study, the CNC diameter of 22 ears (0.66 ± 0.13 mm) with CN hypoplasia was smaller than that of the ears with normal CNCs (1.78 ± 0.10 mm) (*P* = 0.037). Normal ears (*n* = 21) without SNHL had CNC diameters ranging from 1.4 to 2.3 mm. Consequently, we classified CNC diameters <1.4 mm as stenotic.

### CN

When IAM abnormalities are evident in the CT images of patients with severe to profound SNHL, it is important to determine whether the CN is abnormal. However, some factors like high cost have limited the use of MR examinations. Of the 37 patients in this study, 18 received MR examinations. According to Kim et al. [8], the diameter of the CN is greater than that of the superior or inferior branches of the vestibular nerve in 90 % of the unaffected population, and it is also greater than that of the FN in 65 %. In the present study, we defined CN hypoplasia as the CN with its diameter smaller than that of the FN, as determined through oblique sagittal reconstruction of the IAM. CN aplasia was diagnosed when the CN was not detected.

Dysfunctional CN is one of the important causes of congenital SNHL, accounting for 10 % of the children newly diagnosed with SNHL [22]. Sennaroglu et al. reported a case with IP-I malformation with IAM enlargement where the CN was also absent. Adunka et al. [22] reported a case of aplastic CN with a normal IAM, and believed that CN deformity was a result of vascular compression that caused nerve fiber degeneration within the IAM due to cochlear infection or trauma. In the present study, CN hypoplasia was detected with MRI in patients (i.e. case 28 and 36) with either normal or enlarged IAM. In addition, one patient was diagnosed with bilateral IAM stenosis with undetected CN, with the right ear maintaining residual hearing. In this case, it is likely that the CN was too thin to be detected with MRI, or it may have been intermingled with facial or vestibular nerve fibers, thereby rendering it undetectable. Therefore, MRI diagnosis of undetected CN includes only two hearing loss scenarios: (1) CN aplasia presenting as total hearing loss, or (2) CN hypoplasia with residual hearing.

As CI increasingly gains acceptance for the treatment of severe to profound SNHL, inner ear malformations (excluding Michel malformation) are no longer contraindicated for it. However, CN aplasia is a contraindication for CI. Although patients with CN hypoplasia may benefit from CI, the outcome is not as good as in patients with normal CN diameters [23]. Therefore, as a matter of fact, the only critical point to decide a CI would be the normality of CN diameter on MRI, and the IAM and CND criteria are rather useless as compared to this one. However, practically, it is occasionally difficult to define with certainty what is a normal cochlear nerve diameter on MRI. On the other hand, using our criteria, i.e. the diameter of the CN as compared to that of the FN, 35 % of the CNs reported by Kim et al. [8] fall into the “hypoplastic” category in spite of a normal hearing. Moreover, the data presented in Table 1 shows that the CN and CND diameters are not perfectly correlated parameters, namely the CND can appear to be small in spite of an apparently normal diameter of the CN (patient 20, right ear) and vice versa (patient 33, left ear). Both criteria probably represent complementary instead of redundant information. Therefore, it should be combined with the hearing level and imaging results to judge comprehensively. Even so, there is still a need for an improved method for classifying malformations of the IAM, CNC, and CN, which will have significant implications for evaluating the fitness of children with severe to profound SNHL for CI. Based on the findings, we are proposing a new framework, the M(C)ND classification system, for classifying congenital malformations of the IAM, CNC and CN. M, C and N, respectively, represent the IAM, the CNC and the CN to evaluate anatomical variations of them with adding digital (0, 1, 2) representation, as identified by temporal bone CT and MRI reconstructions of oblique sagittal IAM. Practically, one can choose IAM or CNC malformation to record because of their similar implication in this classification system. However, whether an abnormal IAM diameter represents a useful independent prognostic factor cannot be decided based on our data. In fact, in the present series, whenever the IAM was abnormally small (M0 or M1), there was also a small CNC or CN. Additional studies will be necessary to decide if the IAM criterion has to be conserved or can be discarded from our classification. D could be used in combination with previous inner ear classification systems such as Sennaroglu’s to describe whether there is (are) other concomitant inner ear deformitie(s). The D category regarding the inner ear morphology is essential since some cochlear malformations can clearly hinder the outcomes of cochlear implants. For instance, the prognostic value of a cochlear aplasia can certainly not be compared with that of an IP-II (Mondini malformation) in terms of cochlear implant outcome. In this classification system, based on Sennaroglu’s classification, inner ear malformations are divided into two groups in descending order of severity, namely severe (D0) and less severe malformations (D1), with very different prognostic values.

MSCT data in the present study suggest that the magnitude of IAM or CNC stenosis is an indicator of CN aplasia or hypoplasia, and MR examinations must be carried out for further clarification. Compared with the criteria of only a decreased IAM diameter by Casselman et al. [6], our classification system may provide more valuable information for preoperative evaluation and postoperative prognosis of CI. For example, CI is contraindicated for M0, C0, M1N0, and C1N0 patients. Furthermore, our classification system will facilitate the evaluation of post-implantation outcomes for other categories of inner ear and IAM malformations.

Based on the numerous structural malformations of the inner ear, IAM malformations can be classified as single-branch (not combined with other inner ear malformations) or multi-branch (combined with other inner ear malformations). Single-branch malformations include isolated narrowing or enlargement of the IAM that can coincide with absent, narrowed, enlarged, or normal CNC, and with an aplastic, dysplastic, or normal CN. Single-branch malformations also include normal IAM with CN hypoplasia or aplasia [5]. In our study, 18 ears could be categorized as single-branch IAM malformations (D2) and 35 ears as multi-branch (D0, D1). Multi-branch malformations occur more often than single-branch malformations and can occur in any ear [5]. Our study included both simple and complex IAM malformations of both ears (Table 1).

The actual situation could be more complex than we thought it would be. Accordingly, it should be pointed out that our classification system is unable to encompass all possible malformations and may require further revision.

### Clinical correlation

Our classification system for malformations of the IAM, CNC, and CN would standardize the reporting of congenital malformations. This would permit valid preoperative and postoperative comparisons and evaluations for CI.

In our study, four patients who underwent CI were evaluated for M1(C1)N0D0, M2(C1)N1D1 (common cavity), M2(C0)N0D1 (common cavity), and M2(C2)N2D1 (enlarged vestibular aqueduct) with our classification system and follow-up audiological examinations were conducted. No improvement in hearing or speech rehabilitation was shown in patients with M1 or N0 classifications. The outcome of CI for common cavity (M2N0 or C0N0) malformations was worse than for common cavity (M2N1 or C1N1) malformations.

There are reports of patients (classified as M1N0 by our grading system) with residual hearing [24], possibly due to tenuous CN fiber undetectable by MRI. Functional MRI or PET-CT investigations might be applied to evaluate patients prior to CI and may further aid in classifying CN malformations.

## Conclusion

Based on MSCT, IAM or CNC stenosis is highly suggestive of CN hypoplasia or aplasia. MR examinations are needed to refine preoperative and prognostic evaluations of patients with IAM and CNC stenosis who are candidates for CI. Our M(C)ND framework for classifying malformations of the IAM, CNC, and CN is conducive to standardized reporting and may have substantive clinical value.
